# Down-regulation of the let-7i facilitates gastric cancer invasion and metastasis by targeting COL1A1

**DOI:** 10.1007/s13238-018-0550-7

**Published:** 2018-06-01

**Authors:** Yue Shi, Zipeng Duan, Xun Zhang, Xiaotian Zhang, Guoqing Wang, Fan Li

**Affiliations:** 1 Department of Pathogenobiology, The Key Laboratory of Zoonosis, Chinese Ministry of Education, College of Basic Medicine, Jilin University, Changchun, 130021 China; 2 The Key Laboratory for Bionics Engineering, Ministry of Education, Jilin University, Changchun, 130021 China

**Dear Editor,**

Globally, gastric cancer is the most common malignant tumor and the second highest contributor to cancer deaths after lung cancer (Murray et al., 2012). Despite improved success with treatment of early stage gastric cancer (Fuse et al., 2016), the five-year survival rate of advanced staged gastric cancer patients is still low. The aggressive growth characteristics of the tumor and metastasis are key factors responsible for poor overall survival in these patients (Ozkan et al., 2005). Therefore, investigation of the molecular mechanisms that underlie the aggressive behavior of gastric cancers, and identification of potential target genes for therapeutic interventions, is a key imperative.

Aberrant miRNA expression is a key contributor to tumorigenesis in humans (Croce and Calin, 2005; Iorio et al., 2005; Wu et al., 2009; Acunzo and Croce, 2016). Our previous study showed that as a crucial hub gene in gastric cancer, COL1A1 is directly regulated by let-7i miRNA and its high expression levels in gastric cancer have been linked to increased tumor invasiveness (Shi et al., 2015). Downregulation of Let-7i in several cancers was shown to be associated with unfavorable prognosis (Yang et al., 2008; Yang et al., 2013). However, whether let-7i influences progression of gastric cancers is not known.

In the present study, we assessed the let-7i expression level and its effects in gastric cancer samples and cell lines. The binding sites of COL1A1 and let-7i were predicted using bioinformatics software and their regulatory mechanism verified. Further, we also analyzed expression levels of COL1A1 in gastric cancer tissues and cell lines. The results suggest that increased let-7i expression may lead to decrease in proliferative, metastatic and invasive properties of cancer cells.

Expression levels of let-7i were assessed in 40 pairs of gastric cancer tissue specimens and their corresponding adjacent normal tissue samples by qRT-PCR. The result showed that let-7i expression was significantly low in gastric cancer than in normal tissues (Fig. 1A, *P* < 0.001). Further, expression levels of let-7i were lower in gastric cancer cell lines (SGC-7901, MGC-803, AGS, N87) as compared to that in normal gastric epithelial cells GES-1, while no significant difference in this respect was observed in the MKN-45 cell lines (Fig. 1B). A statistically significant association was observed between low expression level of let-7i and T stage (*P* < 0.05; Fig. S1A), and lymph node metastasis (*P* < 0.05; Fig. S1B). The effects of let-7i restoration on regulation of gastric cancer cell vitality and cell proliferation were assessed by transfecting let-7i mimic or miRNA negative control into two human gastric cancer cell lines, SGC-7901 and MGC-803, which have relatively lower levels of let-7i expression. As expected, ectopic let-7i expression markedly suppressed viability of SGC-7901 (*P* < 0.05; Fig. 1C) and MGC-803 cell lines (*P* < 0.05; Fig. S2A) as assessed by use of cell counting kits. Furthermore, over expression of let-7i also reduced proliferation of both SGC-7901 (*P* < 0.05, Fig. 1D) and MGC-803 cells (*P* < 0.05, Fig. S2B), 48 h after transfection, as revealed on colony formation assay. Overexpression of let-7i also reduced invasive and migratory ability of both SGC-7901 (*P* < 0.05, Fig. 1E and 1F) and MGC-803 cells (*P* < 0.05, Fig. S2C and S2D). These findings suggest that let-7i reduced cell viability and proliferative ability and inhibited invasive and migratory properties of gastric cancer cells *in vitro*. SGC-7901 cell lines which stably expressing let-7i and miRNA-control were subcutaneously injected into the dorsal flank of nude mice to evaluate the *in vivo* effects of let-7i on gastric cancer tumor growth. The result showed reduced tumor volume and tumor weight in nude mice with let-7i mimic injection (Fig. 1G–I), which suggests a role of let-7i in modulating *in vivo* gastric cancer progression. In addition, SGC-7901 cells stably expressing let-7i and miRNA-control cells were transplanted through the lateral tail vein to evaluate the effects of let-7i expression on tumor metastasis. Macroscopic observation and histological analysis of the livers showed that the ectopic expression of let-7i significantly inhibited metastasis in organs (Fig. 1J).

**Figure 1:**
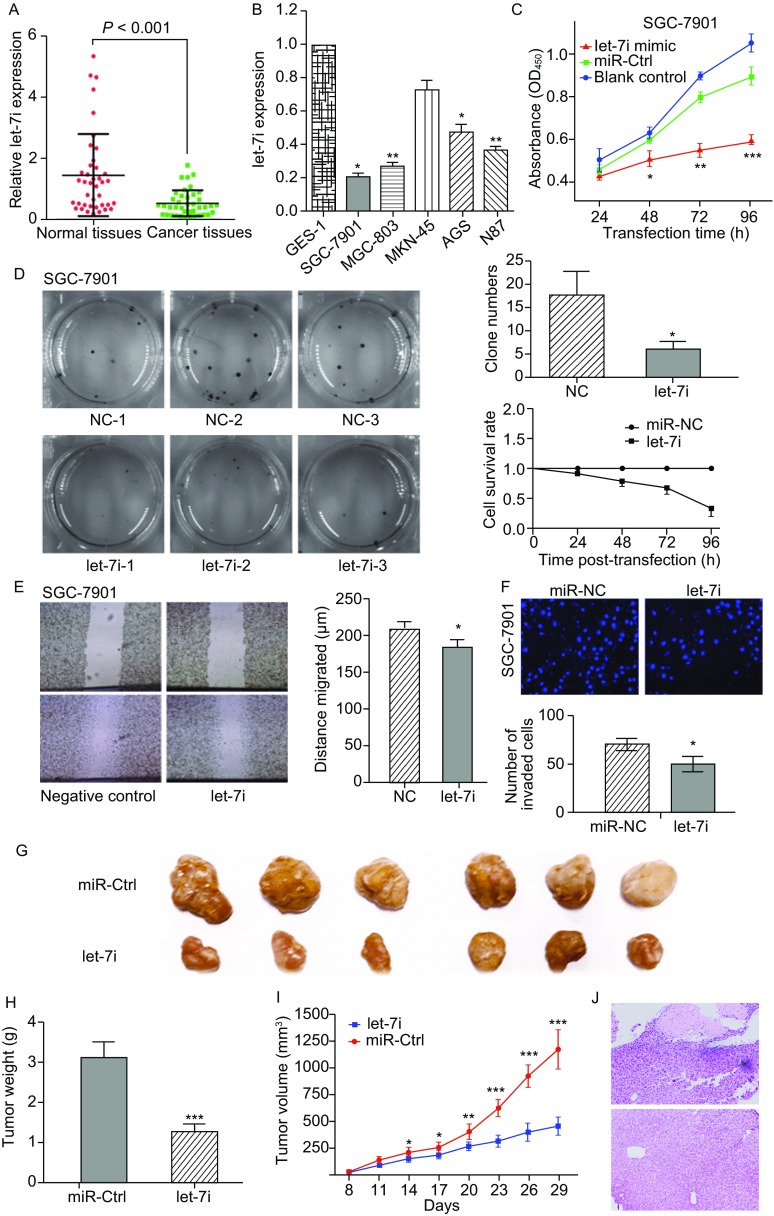
**Downregulation of let-7i expression in gastric cancer tissues and cell lines, effect of ectopic expression of let-7i on tumor cell viability, proliferative properties of SGC-7901, nude mouse xenograft formation and growth after restoration of let-7i expression**. (A) qRT-PCR analysis of let-7i expression in 40 pairs of gastric cancer and their corresponding normal tissues. (B) Let-7i expression in gastric cell lines (SGC-7901, MGC-803, MKN-45, AGS, N87) compared with normal gastric epithelial cells (GES-1), as assessed on qRT-PCR. *P < 0.05 and **P < 0.01. (C) Cell viability assay for SGC-7901. *P < 0.05, **P < 0.01 and ***P < 0.001. (D) Colony formation assay for SGC-7901. *P < 0.05. (E) Wound healing assay for SGC-7901. *P < 0.05. (F) Transwell assay for SGC-7901 *P < 0.05. (G) Nude mouse xenograft assay. (H) Tumor weight formed by the indicated cells. ***P < 0.001. (I) Time-dependent tumor volumes (mm^3^) of miRNA negative control and let-7i mimics mice. *P < 0.05, **P < 0.01, and ***P < 0.001. (J) Pathologic sample and macroscopic observation in livers

We already assessed the expression of COL1A1 in gastric cancer tissues and hypothesized COL1A1 to be one of the target genes of let-7i in gastric cancer cells which mediates its effect through a TF-miRNA co-regulated network identified in our previous study. We reassessed COL1A1 expression in gastric cancer tissue samples and found that level of COL1A1 mRNA increased in gastric cancer tissues compared to the adjacent normal tissues (Fig. 2A). Comparison of COL1A1 mRNA expression with let-7i in gastric cancer exhibited an inverse association (*r* = −0.564, *P* = 0.01; Fig. 2B). We then determined the binding sites of COL1A1 and let-7i by using some specific public bioinformatics tools (TargetScan, miRanda, RNA22 and miRDB). Further, the bioinformatics analysis revealed that COL1A1 served as the direct target of let-7i. RT-PCR and Western blot analysis were performed to analyze the expression of COL1A1 mRNA and protein level in both SGC-7901 and MGC803 cell lines after transfected with let-7i mimic or miRNA-control. COL1A1 expression was significantly decreased in cells which were transfected with let-7i mimic (Fig. 2C and 2D). On bioinformatics analysis, the 3′-untranslated region (3′-UTR) of COL1A1 was found to contain a conserved putative target site for let-7i (Fig. 2E). Luciferase reporter assay showed a significant reduction of luciferase activity in both SGC-7901 and MGC-803 cells after transfection with let-7i mimic as compared to that in the negative control (Fig. 2F). In addition, mutation of the predicted-binding site of let-7i on the COL1A1 3′-UTR rescued the luciferase activity, which indicated that COL1A1 is a direct target gene of let-7i in gastric cancer cells. In addition, the COL1A1 expression vector pWSLV-01-COL1A1 was used to restore COL1A1 expression (Fig. S5A and S5B). Overexpression of COL1A1 rescued the let-7i mediated inhibition of SGC-7901 (Fig. 2G–I) and MGC-803 (Fig. S5C–E) proliferation, migration and invasion. These results suggested COL1A1 to be a direct target of let-7i in gastric cancer cells. The expression of COL1A1 was artificially suppressed in SGC-7901 and MGC-803 cells by using a specific siRNA (Fig. S3A and S3B). On cell counting kit and colony formation assay, knockdown of COL1A1 expression appeared to reduce cell viability and proliferative ability, as compared to that in siRNA control, in both SGC-7901 (Fig. S3C and S3D) and MGC-803 cells (Fig. S4A and S4B). In addition, knockdown of COL1A1 reduced tumor cell invasion and migration capacity of both SGC-7901 (Fig. S3E and S3F) and MGC-803 cell lines (Fig. S4C and S4D). These results were consistent with those observed after up-regulation of let-7i expression.

**Figure 2:**
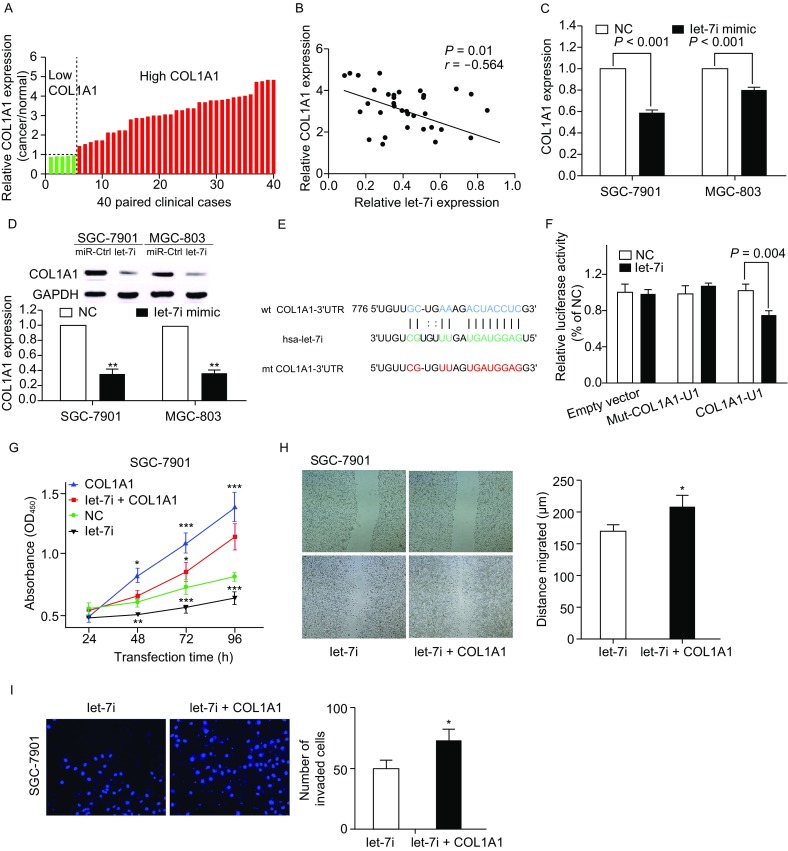
**COL1A1 is the direct target of let-7i in gastric cancer cells**. (A) qRT-PCR analysis of COL1A1 expression in 40 pairs of gastric cancer and corresponding normal tissues. (B) Comparison of let-7i with COL1A1 level in gastric cancer (*r* = −0.564, *P* = 0.01). (C) qRT-PCR for assessment of COL1A1mRNA expression after miRNA-negative control or let-7i mimic transfection into gastric cancer cells. (D) Western blot. Level of endogenous COL1A1 protein after miRNA negative control or let-7i mimic transfection into gastric cancer cells. **P < 0.01. (E) Sequence alignment. Let-7i sequences and its predicted binding site in 3′-UTR of COL1A1. Plasmids contain wild-type or mutant sequences. (F) Luciferase reporter assay. (G) Cell viability of SGC-7901 was assessed after 24, 48, 72 and 96 h transfection with let-7i mimics or let-7i mimics and pWSLV-01-COL1A1 plasmid into gastric cancer cells. *P < 0.05, **P < 0.01 and ***P < 0.001. (H) Wound healing assay for SGC-7901. Tumor cells were transiently transfected with let-7i mimics or let-7i mimics and pWSLV-01-COL1A1 plasmid into gastric cancer cells for up to 24 h. *P < 0.05 (I) Fluorescent images (left) and quantification (right) of invasion level of SGC-7901 after 24 h transfection with let-7i mimics or let-7i mimics and pWSLV-01-COL1A1 plasmid, *P < 0.05

In our previous microarray study, up-regulation of COL1A1 in gastric cancers was significantly associated with invasiveness of gastric cancer (Shi et al., 2015). By applying the TF-miRNA co-regulatory network, we established COL1A1 to be a target gene of let-7i. Thus, we inferred that the expression levels and functional mechanism of COL1A1 might be associated with the regulatory effects of let-7i. Being a recently discovered member of the let-7 family, functional characterization of let-7i is not well-investigated. A recent study showed an association of low expression of let-7i in gastric cancer with tumor cell invasion and lymph node metastasis (Liu et al., 2012). Yang et al. reported an increased susceptibility of ovarian cancer cells to chemotherapy after up-regulation of let-7i (Yang et al., 2008). Blower et al., too, reported increased or decreased sensitivity of tumor cells to chemotherapy drugs after up-regulation or down-regulation of let-7i expression in NCI-60 cancer cell lines, respectively (Blower et al., 2008). In the current study, we observed significant down-regulation of let-7i expression in gastric cancer tissue specimens as compared to that in the corresponding normal tissue specimens. Since, down-regulated let-7i expression was significantly associated with lymph node metastasis and advanced T stage, these results suggest that let-7i may have a suppressive role in progression of gastric cancer. Let-7i was also significantly down-regulated in gastric cancer cell lines (SGC-7901, MGC-803, AGS, N87) as compared to that in normal gastric epithelial cell line GES-1. Let-7i restoration reduced gastric cancer cell viability, migration and invasive properties *in vitro*. Furthermore, restoration of let-7i expression inhibited growth of nude mouse xenografts *in vivo*.

Collagen is an important component of ECM that occurs as collagen types I, II and III in the highest proportions. As a member of the collagen I family, association of COL1A1 with tumor cell proliferation and invasion has been reported in many cancers such as breast, lung and renal cancers (Boguslawska et al., 2015; Grigoroiu et al., 2015; Chai et al., 2016). In our previous study, we demonstrated that up-regulated expression of COL1A1was closely associated with invasive properties of gastric cancer cells. In this study, we found that COL1A1 expression was inversely associated with let-7i levels in gastric cancer tissues. Level of COL1A1 mRNA and protein expression was significantly reduced after ectopic expression of let-7i in gastric cancer cells. Luciferase activity assay further demonstrated that COL1A1 was a direct target of let-7i. Moreover, knockdown of COL1A1 in gastric cancer cells curbed the proliferative, migratory and invasive ability of cancer cells, which was similar to the results obtained with artificial upregulation of let-7i. Moreover, the restoration of COL1A1 expression in cells stably expressing let-7i was able to counteract the inhibitory effects of let-7i in gastric cancer cells. Taken together, our findings provide strong evidence of COL1A1 as being a direct and functional target of let-7i.

In summary, the present study provides strong evidence of suppressive effects of let-7i on gastric cancer development and progression. Ectopic expression of let-7i successfully suppressed multiple malignant biological characteristics including inhibition of tumor cell viability and reduction of migration and invasion *in vitro* and tumor xenograft growth *in vivo*. Our further experiments revealed that overexpressed COL1A1 was a direct target of let-7i in gastric cancer cells. Knockdown of COL1A1 in gastric cancer cells produced an anti-proliferative, migratory and invasive reduced effect, which was similar to the results when let-7i up-regulation. These results suggest a potential application of let-7i as a diagnostic and therapeutic target for gastric cancers. Future studies will seek to confirm the functional mechanisms of let-7i in gastric cancer.

## Footnotes

This work was supported by grants from National Natural Science Foundation of China (Grant Nos. 81320108025, 81472662 and 81672109), foundation of Jilin Province Science and Technology Department (172408GH010234983), and Jilin University-Xinjiang Medical University joint research project.

Yue Shi, Zipeng Duan, Xun Zhang, Xiaotian Zhang, Guoqing Wang and Fan Li declare that they have no conflict of interest.

All procedures followed were in accordance with the ethical standards of the responsible committee on human experimentation (institutional and national) and with the Helsinki Declaration of 1975, as revised in 2000 (5). Informed consent was obtained from all patients for being included in the study.

All institutional and national guidelines for the care and use of laboratory animals were followed.

## Electronic supplementary material

Below is the link to the electronic supplementary material.

Supplementary material 1 (PDF 614 kb)
