# Supplementary material for: Down-regulation of the let-7i facilitates gastric cancer invasion and metastasis by targeting COL1A1
Source: Protein Cell. 2018 Jun 1;10(2):143–8. doi: 10.1007/s13238-018-0550-7 (PMC6340894; doi:10.1007/s13238-018-0550-7)
Supplement: Supplementary file 1 — Supplementary material 1 (PDF 614 kb) [file 13238_2018_550_MOESM1_ESM.pdf]

Materials and methods

Samples from gastric cancer patients

We collected 40 samples of gastric cancer tissue and corresponding distant normal tissue samples from gastric cancer patients between December 2008 and May 2011 at the Jilin University (Changchun, China). The study was approved by the Ethics Committee at the School of Basic Medical Sciences, Jilin University; written informed consent was obtained from each patient. All patients were diagnosed with gastric cancer and had undergone radical gastric resection. Data on clinicopathological parameters were obtained from their medical records and are summarized in Table S1. All tissues were stored in liquid nitrogen within 10 min of their collection.

Cell lines and cultures

Human gastric cancer cell lines SGC-7901, MGC-803, MKN-45, AGS and N87 and normal gastric epithelial cell line GES-1 were obtained from American Type Culture Collection, ATCC (Manassas, VA, USA) and were maintained in RPMI 1640 medium (Invitrogen, Carlsbad, CA, USA) supplemented with 10% FBS (Invitrogen) at 37°C in a 5% CO_2 atmosphere.

RNA isolation and qRT-PCR

Total RNA was isolated from tissue samples and cell lines using Trizol reagent (Invitrogen); miRNA was isolated using miRcute miRNA isolation kit (Tiangen, Beijing, China) according to the manufacturer's instructions. RNA concentration was measured using the Epoch Multi-volume Spectrophotometer System (BioTek, Vermont, USA). Further, the RNA samples were reverse-transcribed into cDNA using a PrimeScript RT reagent Kit (TaKaRa Otsu, Shiga, Japan) and miRcute miRNA Fist-Strand cDNA synthesis kit (Tiangen, Beijing, China). qPCR was performed using SYBR Premix ExTaq (TaKaRa) and miRcutemiRNAqPCR detection kit (SYBR Green) (Tiangen). β-actin and U6 were used to normalize the expression levels of mRNA and miRNA. The primers of β-Actin were: 5'-CTGGAACGGTGAAGGTGA CA-3 and 5'-AAGGGACTTCCTGTAACAATGCA-3'，

The primers for COL1A1 were: 5'-GAGGGCCAAGACGAAGACATC-3' and 5'CAGATCACGTCATCGCACAAC-3'. The primers of let-7i were 5'-ATGGTTCGTGGGTGAGGTAGTAGGTTGT-3' and 5' GCAGGGTCCGAGGTATTC-3'. The primers of U6 were 5'-CTCGCTTCGGCAGCACA-3' and 5' AACGCTTCACGAATTTGCGT-3'. qPCR was performed using ABI 7300 system and the data was analyzed using the $2^{\Delta\Delta CT}$ method.

Protein extraction and Western blot

Total cellular protein was extracted from tissue samples and cell lines using Cell lysis buffer for Western and IP (Beyotime, Shanghai, China). These protein samples (20 μg each) were subjected to sodium dodecyl sulfate-polyacrylamide gel electrophoresis (SDS-PAGE) and transferred onto PVDF membranes (Millipore, Bedford, MA, USA). The membranes were

blocked in 5% skimmed milk and then incubated with primary and secondary antibodies respectively. The immunoblots were visualized using Gene gnome Syngene bio imaging (Syngene, Cambridge, UK) and protein levels were normalized to GAPDH and expressed with fold changes. A primary anti-COL1A1 antibody was obtained from Santa Cruz Biotechnology (#sc-8784, Santa Cruz, CA, USA) and was used to conduct Western blot and immunohistochemical examination, while GAPDH antibody and secondary antibodies were purchased from Beyotime (Shanghai, China).

Gene transfection

Let-7i mimic, miRNA mimic control, siRNA against COL1A1 and siRNA control were synthesized by Ribobio (Guangzhou, China) and transfected into gastric cancer cell lines using Lipofectamine RNAiMAX (Invitrogen, USA) according to the manufacturer's instructions. The pWSLV-01-COL1A1 plasmid was constructed and transfected into gastric cancer cell lines using Lipofectamine LTX (Invitrogen, USA).

Luciferase reporter assay

A wild-type 3'-UTR fragment of COL1A1 cDNA was amplified using PCR and cloned into *Xba*I and *Sac*I site of pmirGLO dual-luciferase miRNA target expression vector (Promega, Madison, WI, USA) and named as wtCOL1A1-3'-UTR. The mutant variant of COL1A13'-UTR was generated based on wtCOL1A1-3'-UTR by mutating 12 nucleotides that potentially bind to let-7i and named as mtCOL1A1-3'-UTR. These vectors (wtCOL1A1-3'-UTR or wtCOL1A1-3'-UTR together with let-7i mimic or miR-ctrl) were transiently transfected into gastric cancer SGC-7901 and MGC-803 cells using Lipofectamine 2000 reagent (Invitrogen). Luciferase activity was detected after 48 h using Dual-Glo luciferase assay system (Promega) using Synergy H1 Multi-Mode Microplate Reader (BioTek, Vermont, USA). Luciferase activity ratios are expressed as firefly luciferase values / renilla luciferase values, after normalizing them to the control plasmid.

Cell proliferation and colony formation assay

Tumor cell viability was assessed using Cell Counting Kit-8 (CCK8) kit (Dojindo Molecular Technologies, Kumamoto, Japan) according to the manufacturer's protocol after gene transfection. Experiments were repeated at least three times with similar data. Colony formation assay was performed after transfection of let-7i mimic or siRNACOL1A1 into SGC-7901 and MGC-803 cell lines to assess proliferation capacity of gastric cancer cells, colony measurement was made after 3 weeks of culture.

Wound healing and tumor cell invasion assay

Tumor cell migration capacity was assessed using wound healing assay in 24-well plates, SGC-7901 and MGC-803 cells were transfected with let-7i mimic or siRNACOL1A1. Post 48 h scratches in plate were made by tips, the blank areas in plate at 0 h and 24 h were measured, respectively, the difference between the two was the cell migration area. Tumor cell invasion capacity was assessed using Transwell chamber (Corning, Corning, NY, USA)

in 24-well plates. In brief, SGC-7901 and MGC-803 cells were transfected with let-7i mimic or siRNACOL1A1; after 24h, cells were resuspended in a serum-free RPMI 1640 medium and seeded onto the upper chamber with or without Matrigel coated membrane (BD Biosciences, San Jose, CA), while the lower chamber was filled with a fresh medium containing 10% FBS. Cells migrating and invading into the lower surface of the chambers were fixed in paraformaldehyde and stained with DAPI (4',6-diamidino-2-phenylindole) and examined reviewed and photographed under a fluorescence microscope at ×200 magnification in three random fields.

Nude mouse gastric cancer cell xenograft model

Twenty male BALB/c nude mice were purchased from Guangdong Medical Laboratory Animal Center (Foshan, China). The experimental protocol was approved by the Animal Care and Use Committee at the College of Basic Medicine, Jilin University. Let-7i mimic and miRNA mimic control were transfected into gastric cancer cell lines using Lipofectamine RNAiMAX (Invitrogen, USA) according to the manufacturer's instructions. Single-cell suspension（1×10^6 cells/ml）was made 48h post transfection. BALB/c nude mice were randomly divided into two groups: miRNA controls and let-7i mimic groups. The dorsal flanks of nude mice were subcutaneously injected with SGC7901/miR-Ctrl and SGC7901/let-7i (1×10^6 cells per mouse). Tumor size was examined with vernier caliper once every 3 days beginning 8 days after the injection. Tumor volume was calculated by the formula: tumor volume = length × width^2 × 0.5. All mice were sacrificed thirty days after transplantation of tumor cells; tumor xenografts were removed and weighed. Moreover, the livers were removed, paraffin-embedded, and subjected to hematoxylin-eosin staining and observed by microscope.

Statistical analysis

All statistical analyses were performed using SPSS version 18.0 (WPSS Ltd, Surrey, UK) and GraphPad Prism 6 software (GraphPad Software, Inc., CA, USA). The results are presented as mean ± Standard deviation. Relative quantification of mRNA and miRNA expression level was calculated with the $2^{\Delta\Delta CT}$ method. The difference in levels of mRNA, miRNA or protein expression between gastric cancer and corresponding normal tissues was evaluated using the nonparametric Mann-Whitney U-test. Association of let-7i expression with clinicopathological parameters was assessed using Chi-square test or Fisher's exact probability test. For *in vitro* experiments, Student's *t*-test was used to assess between-group differences. Comparisons between multiple groups were assessed using one-way Analysis of Variance (ANOVA). All *P*-values were two sided; $P < 0.05$ was considered to be statistically significant.

Table S1 Clinicopathological characteristics of the study samples

Case	Clinical parameters

	Gender	*Age*	*Differentiation status*	*TNM stage*
1	Male	56	Poorly differentiated	T3N2M1
2	Male	68	Moderately differentiated	T3N1M0
3	Female	58	Poorly differentiated	T3N3M0
4	Male	57	Poorly differentiated	T3N3M0
5	Female	56	Poorly differentiated	T3N1M0
6	Male	66	Moderately differentiated	T3N3M0
7	Male	57	Moderately differentiated	T3N2M0
8	Female	43	Poorly differentiated	T3N2M0
9	Male	56	Poorly differentiated	T3N2M0
10	Male	63	Poorly differentiated	T4N3M0
11	Female	77	Moderately differentiated	T2N0M0
12	Male	63	Poorly differentiated	T3N3M0
13	Female	69	Moderately differentiated	T3N2M1
14	Male	62	Moderately differentiated	T1N0M0
15	Female	47	Poorly differentiated	T3N1M0
16	Male	77	Moderately differentiated	T3N1M0
17	Male	90	Poorly differentiated	T3N2M1
18	Female	48	Poorly differentiated	T3N3M0
19	Male	70	Poorly differentiated	T4N3M0
20	Male	45	Moderately differentiated	T1N0M0
21	Female	39	Poorly differentiated	T3N1M1
22	Female	48	Moderately differentiated	T3N3M0
23	Female	59	Moderately differentiated	T2N0M0
24	Female	64	Moderately differentiated	T3N2M1
25	Female	39	Moderately differentiated	T3N1M1
26	Male	75	Poorly differentiated	T4N3M0
27	Male	60	Poorly differentiated	T3N3M0
28	Male	81	Moderately differentiated	T4N3M0
29	Female	69	Poorly differentiated	T3N3M0
30	Male	53	Moderately differentiated	T2N1M0
31	Male	70	Poorly differentiated	T4N2M1
32	Male	73	Poorly differentiated	T4N3M1
33	Male	74	Moderately differentiated	T3N1M0
34	Male	54	Moderately differentiated	T4N3M0
35	Male	66	Moderately differentiated	T3N3M1
36	Male	65	Poorly differentiated	T3N1M0
37	Female	77	Moderately differentiated	T3N2M1
38	Female	73	Poorly differentiated	T3N3M1
39	Male	64	Moderately differentiated	T3N1M0
40	Male	51	Poorly differentiated	T3N3M1

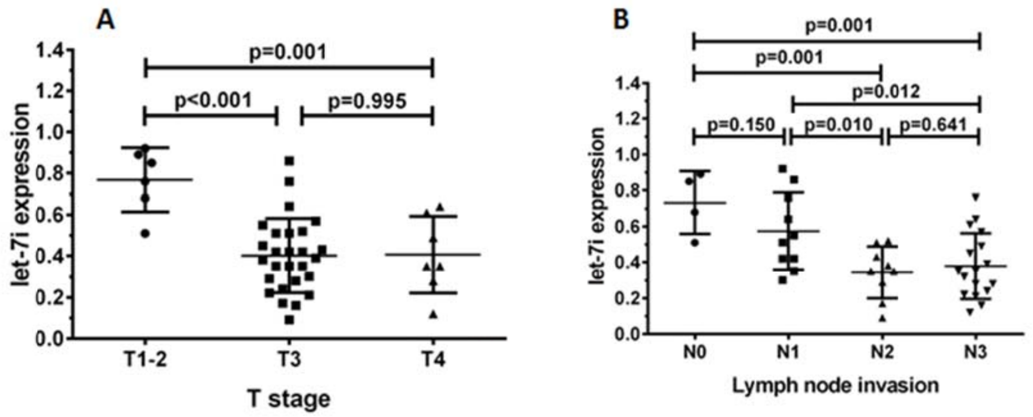

Figure S1. Correlation of let-7i expression with invasive ability and lymph node metastases in gastric cancer. (A) let-7i expression was determined in different tumor T stages (depth of cancer invasion), including 5 cases of T1-2 (mucous and muscular layer), 28 cases of T3 (serosal layer), and 7 cases of T4 (whole layer). (B) let-7i expression was measured in different N stages (lymph node metastases) of gastric cancer (N0 = 4, N1 = 10, N2 = 9, N4 = 17).

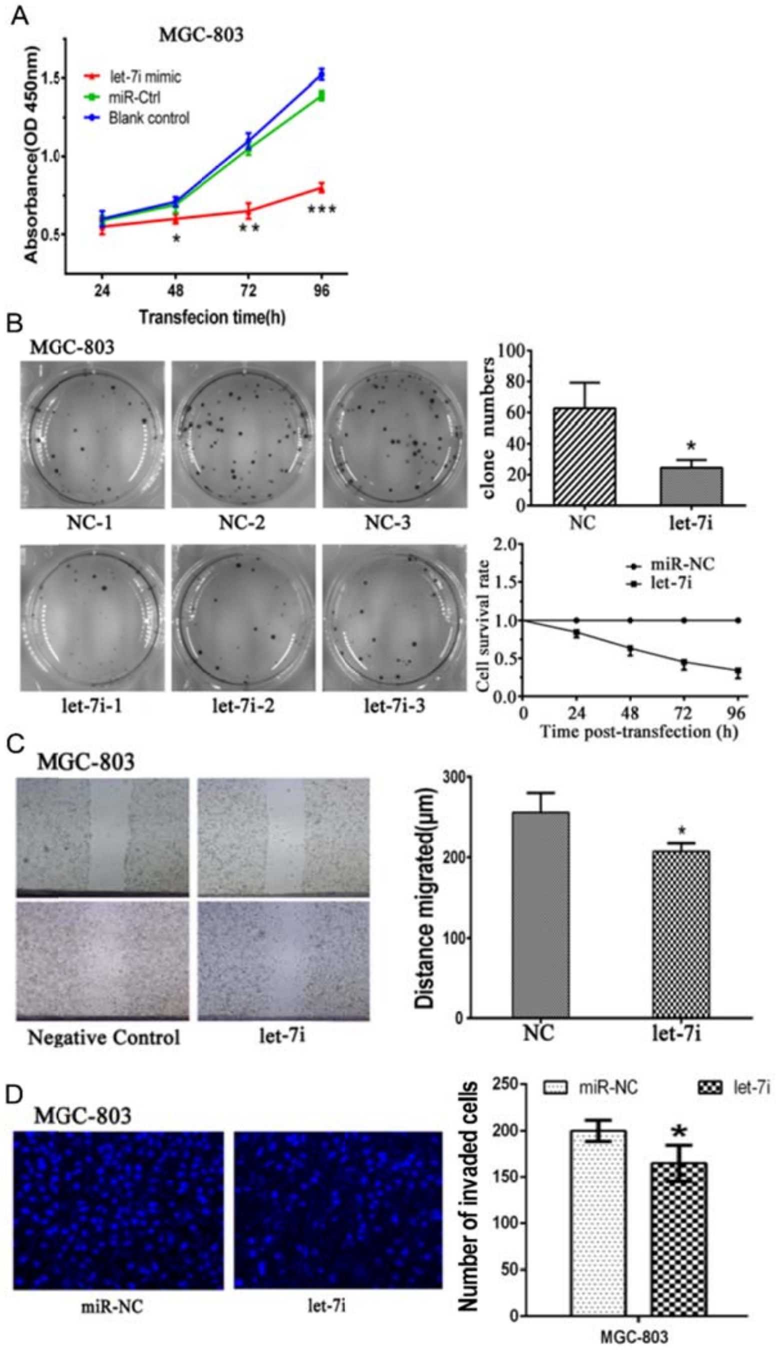

Figure S2. Effect of ectopic expression of let-7i on tumor cell viability, proliferative, migratory and invasive properties of MGC-803 cells

(A) Cell viability assay for MGC-803. *$P < 0.05$, **$P < 0.01$ and ***$P < 0.001$. (B) Colony formation assay for MGC-803. *$P < 0.05$. (C) Wound healing assay for MGC-803. *$P < 0.05$. (D) Transwell assay for MGC-803. *$P < 0.05$.

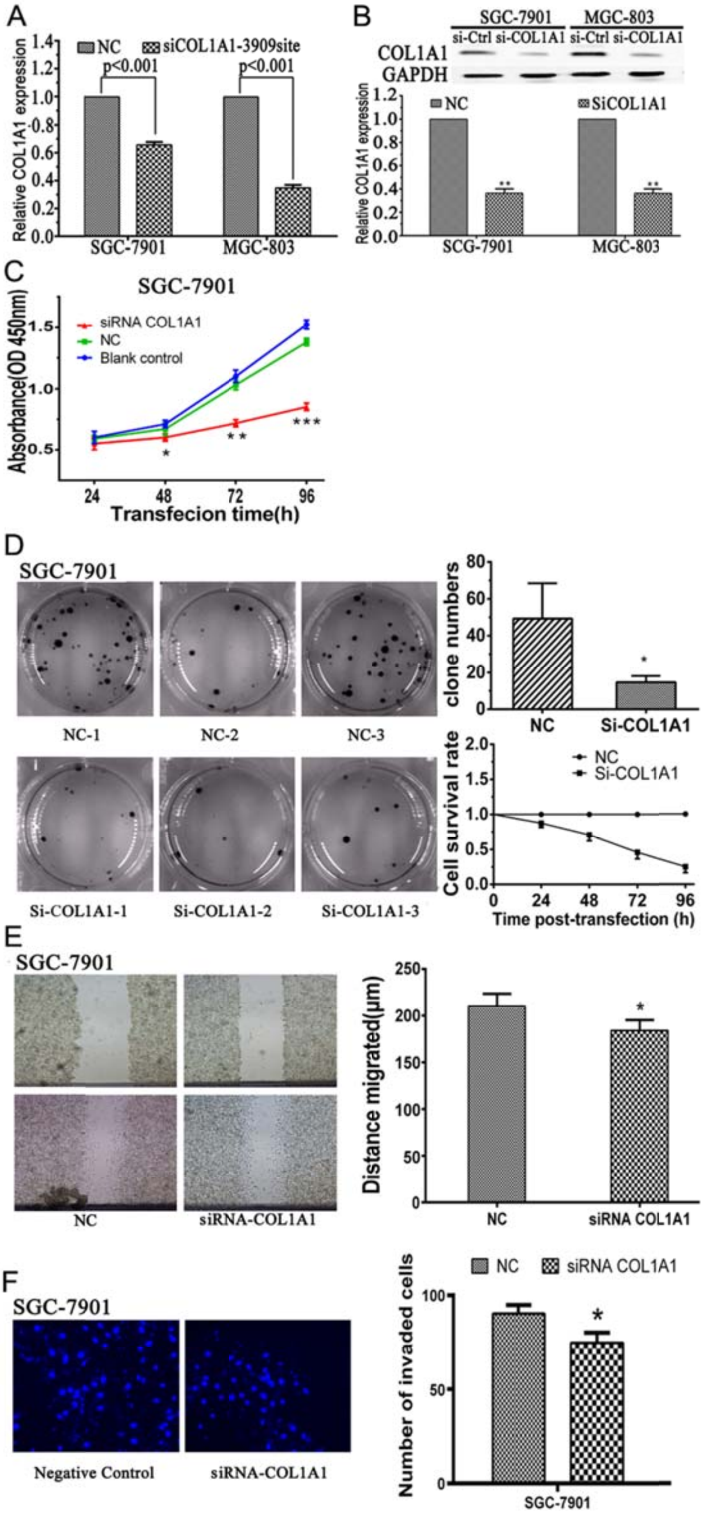

Figure S3. Effects of COL1A1 knockdown on tumor cell viability, proliferative migratory and invasive properties of SGC-7901 cells. (A) RT-PCR for assessment of COL1A1 mRNA after transfection with negative control or COL1A1 siRNA into gastric cancer cells. (B) Western blot. Level of COL1A1 protein was assessed after transfection with negative control or COL1A1 siRNA into gastric cancer cells. (C) Cell viability assay for SGC-7901. *P < 0.05, **P < 0.01 and *** P < 0.001. (D) Colony formation assay for SGC-7901. *P < 0.05 and **P < 0.01. (E) Wound healing assay for SGC-7901. *P < 0.05 (F) Transwell assay for SGC-7901. **P* < 0.05.

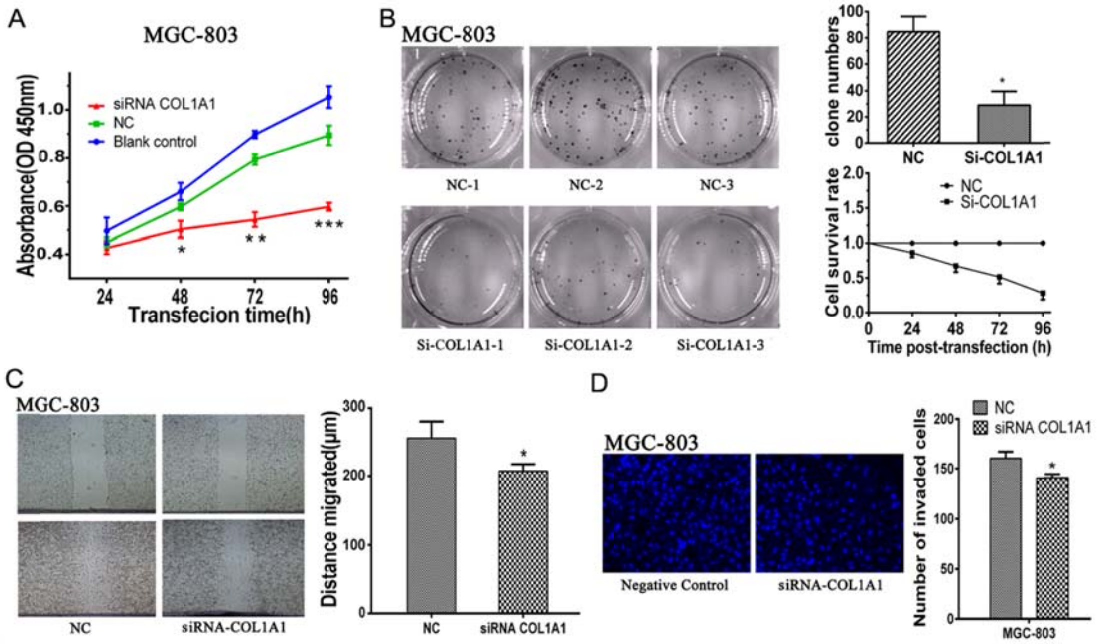

Figure S4. Effects of COL1A1 knockdown on tumor cell viability, proliferative migratory and invasive properties of MGC-803 cells.

(A) Cell viability assay for MGC-803. *P < 0.05, **P < 0.01 and ***P < 0.001. (B) Colony formation assay for MGC-803. *P < 0.05. (C) Wound healing assay for MGC-803. *P < 0.05. (D) Transwell assay for MGC-803. *P < 0.05.

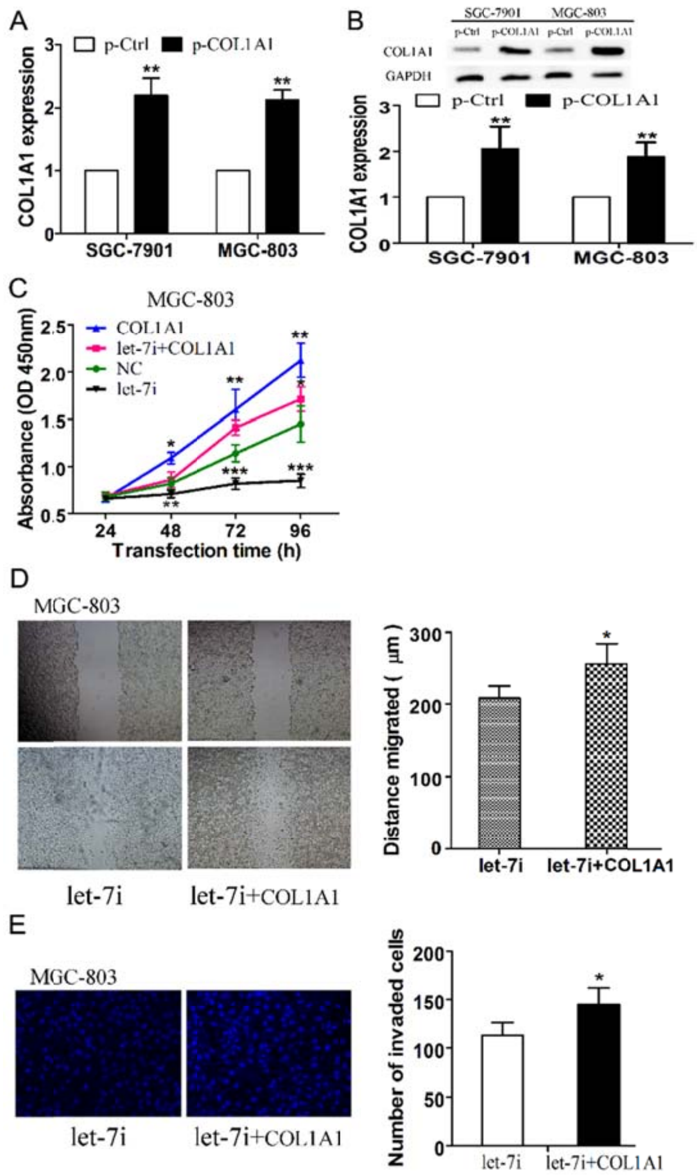

Figure S5. Effects of COL1A1 restoration on gastric cancer MGC-803 cells. Tumor cells were transiently transfected with let-7i mimics or let-7i mimics and pWSLV-01-COL1A1 plasmid and then subjected to different assays.

(A) RT-PCR. Level of COL1A1 mRNA was assessed by RT-PCR after transfection with pWSLV control or pWSLV-01-COL1A1 into gastric cancer cells. (B) Western blot. Level of COL1A1 protein was assessed after transfection with pWSLV control or pWSLV-01-COL1A1 into gastric cancer cells. (C) Cell viability CCK8 assay for MGC-803. *p < 0.05, **p < 0.01 and ***p < 0.001. (D) Wound healing assay for MGC-803. *P < 0.05 (E) Tumor cell invasion assay. Fluorescent images (left) and quantification (right) of invasion level of MGC-803 cells after 24 h transfection with let-7i mimics or let-7i mimics and pWSLV-01-COL1A1 plasmid. *p < 0.05.
